# Lifestyle Interventions for People with, and at Risk of Type 2 Diabetes in Polynesian Communities: A Systematic Review and Meta-Analysis

**DOI:** 10.3390/ijerph15050882

**Published:** 2018-04-28

**Authors:** Dorothy W. Ndwiga, Freya MacMillan, Kate A. McBride, David Simmons

**Affiliations:** 1 School of Science and Health, Western Sydney University, Penrith 2751, Australia; f.macmillan@westernsydney.edu.au; 2 School of Medicine, Western Sydney University, Penrith 2751, Australia; k.mcbride@westernsydney.edu.au (K.A.M.); da.simmons@westernsydney.edu.au (D.S.); 3 Translational Health Research Institute, Western Sydney University, Penrith 2751, Australia

**Keywords:** type 2 diabetes, Polynesian people, diabetes prevention, lifestyle intervention, diet, physical activity

## Abstract

There is evidence that lifestyle intervention among Polynesian people can reduce diabetes incidence and complications, but this evidence has not been systematically reviewed. The aim of this study was to systematically review the efficacy of lifestyle interventions, targeting the prevention and management of type 2 diabetes among Polynesian people. MEDLINE, Web of Science, Cochrane Library, and Embase were searched to find randomised controlled trials (RCTs) and pre-post studies. Eight studies (four RCTs and four pre-post studies) with 1590 participants met the inclusion criteria. The data on health outcomes that was reported in these studies included blood pressure, body mass index, waist circumference, weight, and glycated haemoglobin (HbA1c). The meta-analyses showed that the interventions had resulted in statistically significant reductions in systolic blood pressure (SBP) across four of the studies (WMD, −9.93 mmHg; 95% Cl, −10.77 to −9.09; and *p* < 0.00001). However, the effects on weight across five of the studies (WMD, −1.15 kg; 95% Cl, −2.80 to 0.51; *p* = 0.18) and the HbA1c levels across two of the studies (WMD, −0.38%; 95% Cl, −1.15 to 0.39; and *p* = 0.33) were not statistically significant. This review provides evidence that lifestyle interventions may be effective in achieving modest reductions in SBP in Polynesian people. Further research is needed to fully assess the effectiveness of these interventions in this population long-term.

## 1. Introduction

Type 2 diabetes is one of the leading causes of morbidity and premature death worldwide, and is particularly prevalent in certain populations [1,2,3,4,5]. Pacific People are some of the worst that are affected by diabetes, with prevalence continuing to rise at a much faster rate compared with other ethnic groups [5,6,7]. Pacific People also suffer disproportionately from diabetes-related complications as well as higher rates of avoidable hospital admissions, compared with other groups [4,8,9,10,11]. The high prevalence of diabetes among Pacific People is largely because of the high rates of individuals that are overweight and obese [12], with more than 75% of Pacific People estimated to fall into the overweight (≥25 kg/m^2^) or obese BMI (≥30 kg/m^2^) categories [2,9,13]. Higher BMI cut points ≥26 kg/m^2^ and ≥32 kg/m^2^ for the classification of overweight and obesity for Pacific People have been recommended because of their higher lean mass [2,9,14].

In addition to the health impacts of diabetes on individuals, along with diabetes prevalence being expected to rise from 382 to 592 million by the year 2035 [15], there is a large and growing economic burden on health care systems from the condition [16]. Diabetes currently costs over $14 billion annually in Australia alone [17,18]. Most type 2 diabetes cases are preventable by following a healthy lifestyle [5,9,19,20,21], with clinical trials revealing that for every kilogram of weight lost there is a 16% reduction in diabetes risk [22]. Adopting a healthier diet and increasing physical activity can also reduce and/or prevent the progression of type 2 diabetes by up to 58% in people with impaired glucose tolerance [23,24]. Additionally, in those with diabetes, management of the condition can also be improved by following a healthy lifestyle (alongside any medication), consequently reducing the risk of developing complications from diabetes, such as kidney disease, blindness, nerve damage, and blood vessel damage [7].

In response to the diabetes epidemic, community-based lifestyle interventions have been recommended in order to prevent and manage type 2 diabetes in ethnic minority groups [5,6,12,22]. These interventions need to be culturally appropriate, acceptable, and useable by the target population so as to have a lasting impact [4,25]. Although several intervention studies in Pacific communities have been published, their effects have been contradictory, and we will now undertake a systematic review in order to provide an assessment of the overall impact of these studies on physical health outcomes, such as glycated haemoglobin (HbA1c), blood pressure (BP), weight, and waist circumference) and on the psychological health outcomes.

## 2. Research Design and Methods

### 2.1. Study Eligibility

Randomised controlled trials (RCTs) and pre-post studies which assessed the effectiveness of lifestyle interventions, with the aim of reducing diabetes risk factors or managing diabetes in Polynesians (ethnic group of the Polynesia region in the Pacific Islands), were included. The study eligibility criteria and the search strategy were based on population, intervention, comparison, and outcome (PICO) guidelines for systematic reviews [26]:

*Population:* Polynesian adults (≥18 years) with type 2 diabetes or at risk of developing type 2 diabetes (for example those with obesity, family history of diabetes, and/or impaired glucose tolerant individuals). Studies with mixed ethnicities were included, only if ≥97% of the participants were of Polynesian descent. This arbitrary proportion was used in order to ensure that the intervention was genuinely focused on a Polynesian community.

*Intervention:* Lifestyle intervention, including diet and/or physical activity, lasting ≥3 months (this minimum duration was taken to reflect the time for a complete turnover of HbA1c).

*Comparisons:* Waitlist or usual care (no diet or physical activity support) for both RCTs and pre-post studies.

*Outcomes:* Outcome measures included any physical health outcomes (e.g., HbA1c, BP, and weight) or psychological (e.g., diabetes knowledge) health outcomes.

Peer-reviewed journal articles that were published in English were included. No limit on the publication date was set. Studies were excluded if the participants had other types of diabetes (not type 2 diabetes), were from mixed ethnicities/populations (if ≥3% participants were from non–Polynesian communities), and if full texts were not available.

### 2.2. Search Strategy

The MEDLINE (EBSCOhost), Web of Science (Clarivate), Embase (Ovid), and Cochrane Library (Wiley) databases were searched. The supporting information (Table S1) illustrates the search strategy that was used in Embase. The search strategy was specifically tailored for each database and used a combination of synonyms, which were related to the following keywords, namely: Pacific People (population), diet and/or physical activity intervention (intervention), type 2 diabetes and/or diabetes prevention (condition), and RCT or pre-post study (design). All of the authors reviewed the search strategy.

Database searches were conducted in May 2017 and a final updated search was conducted in December 2017 to ensure no new publications were omitted. Reference lists of included articles were also searched for eligible studies. One researcher (Dorothy W. Ndwiga) initially assessed the relevance of all of the titles and abstracts, based on the inclusion criteria. Two researchers (Freya MacMillan and Kate A. McBride) independently screened 10% of each of the returned references. Further screening, at the full-paper level, was undertaken by one researcher (Dorothy W. Ndwiga), with independent screening also having been undertaken by three researchers on a portion of each of all papers (Freya MacMillan, Kate A. McBride, and David Simmons).

### 2.3. Data Extraction and Assessment of Risk of Bias

One reviewer (Dorothy W. Ndwiga) extracted data on the studies’ and participants’ characteristics, intervention details, inclusion criteria, study outcomes, and findings. The risk of bias for the selected studies was assessed according to the Cochrane collaboration’s tool for assessing the risk of bias in randomised trials [27] and the Risk of Bias in Non-Randomised Studies of Interventions (ROBINS-1) assessment tool for pre-post studies [28]. Independent reviewers extracted data from a portion of all of the included papers (Freya MacMillan, Kate A. McBride, and David Simmons). The authors were contacted where possible. Any discrepancies were resolved through discussion, until a consensus was met. The RCT Risk of Bias tool included criterion for assessing the selection bias, detection bias, attrition bias, and reporting bias, with each category rated as either a low risk, high risk, or unclear risk of bias. The blinding of participants to the intervention (performance bias) was not included as a validity criterion, as this was not possible in lifestyle intervention research (i.e., participants knew if they were receiving an intervention or not). The ROBINS-1 tool assessed the selection bias, confounding bias, attrition bias, bias because of the measurement of outcomes and classification of interventions (detection bias), and bias in selection of the reported results (reporting bias), with each category being rated as low risk, moderate risk, serious risk, critical risk, or no information.

### 2.4. Data Synthesis and Statistical Analysis

The data that was included in the meta-analyses were analysed using the Review Manager (Revman version 5.3, The Nordic Cochrane Centre, The Cochrane Collaboration, Copenhagen, Denmark). The weighted mean difference (WMD) and 95% confidence intervals (95% Cl) were calculated from either the difference in mean and standard deviation (SD) of the study outcomes, before and after the intervention in the intervention and the control group, or by the end of intervention mean and SD in both groups. Where SD was not directly reported, it was calculated from the standard error (SE), using the following formulae: SD = SE × √n. A random effects model was used to summarise the pooled WMD. Chi-square and I^2^-index tests were used to examine the statistical heterogeneity among the studies that were included in the meta-analyses. The publication bias was assessed by visually inspecting the funnel plots.

For studies with two intervention groups, the mean ± SD data were entered for each intervention group and the sample size for the control group was halved for each comparison. Where insufficient outcome data were reported across studies that were to be included in the meta-analysis, a narrative synthesis of the impacts of interventions on the outcomes was conducted.

## 3. Results

### 3.1. Identification of Studies

**Figure 1:**
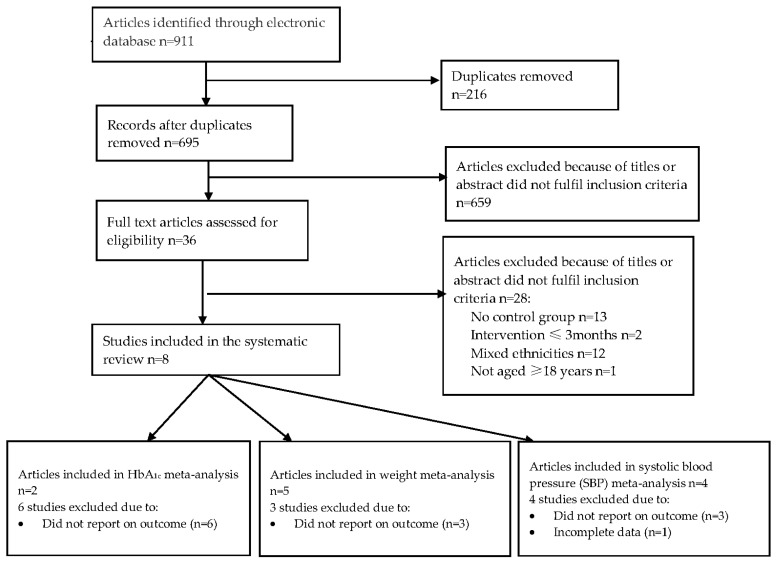
Flow diagram of the study selection.

Database searching retrieved 911 citations. Of these, 877 were excluded at title and abstract stage (Figure 1). There were 36 full texts that were reviewed, with 28 of these excluded because the participants were not Polynesian or because they included <97% of the Polynesian population mixed population (*n* = 12), there was no control/comparison group (*n* = 13), they included participants with other forms of diabetes other than type 2 diabetes (*n* = 1), or the intervention lasted ≤3 months (*n* = 2). A list of studies that were excluded at the final stage of screening and the reasons for exclusion are summarised in Supplementary Table S4.

### 3.2. Participants and Study Characteristics

The total sample size across the included studies was 1590. There was great variability in both the number of participants in each study, which ranged from 55 [29] to 471 [30], with four studies that had more than 210 participants [12,30,31,32], as well as the length of the intervention duration, which ranged from 12 weeks [29] to 2 years [12,32]. Of the four RCTs, only one included a sample size calculation, however it did not reach the target [31]. Only one study included the intention to treat the analysis for missing data [29]. The age and gender (where available) and study characteristics for each study are reported in Table 1. There were eight articles [12,29,30,31,32,33,34,35] that reported on seven unique interventions (one intervention was used in two different target groups—in Tongan [12] and Samoan [33] communities) and met the inclusion criteria. Of these, four were RCTs [29,31,34,35], and four were pre-post studies [12,30,32,33]. All eight of the papers were published between 2001 and 2017. Six studies were conducted in New Zealand [12,30,32,33,34,35] and two were conducted in the United States of America [29,31]. Most of the studies had data collected at baseline and immediately post-intervention, except one [30], where data were collected at baseline and after 8, 16, and 24 weeks.

Two studies focused on the prevention of type 2 diabetes [32,35], two studies focused on the management of diabetes [31,34], and the remaining four studies focused on both the prevention and management of diabetes (participants with and without a known diabetes diagnosis) [12,29,30,33]. Seven studies, which were included in this review, had one intervention and one control group [12,29,30,31,32,33,34], two of which had both intervention and control groups that received diabetes education packages following the baseline assessment [31,34]. The remaining study had two intervention groups that received different diet interventions [35]. Six studies focused on the effectiveness of the combination of diet and physical activity interventions, one of which consisted of providing information only [34], whilst the remaining studies’ participants received the combined practical assistance of changing their diet and physical activity [12,30,31,32,33]. One study focused on physical activity only [29], and one focused solely on diet [35]. All of the studies reported to have incorporated culturally relevant lifestyle messages, including the incorporation of traditional foods and activities.

### 3.3. Risk of Bias of the Included Studies

Tables S2 and S3 summarise the risk of bias within the included RCTs and pre-post studies, respectively. After independently assessing and scoring the risk of bias of the included articles, the researchers (Dorothy W. Ndwiga, Freya MacMillan, and Kate A. McBride) had an 88% similarity to the assessment of risk of bias. All of the studies had explored the differences in participant characteristics between the intervention and control groups at baseline, however none had reported any significant differences. Only one RCT had reported on the method of random assignment [31], and all of the included studies were of a high risk of bias, because of the lack of blinding of the assessors and/or participants, which might have been impractical in a lifestyle intervention.

### 3.4. Impacts of Interventions

#### 3.4.1. Meta-Analyses Findings

**Figure 2:**
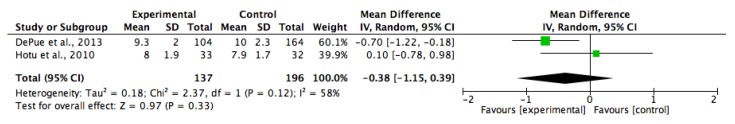
Forest plot showing the mean glycated haemoglobin (HbA1c) (%) change and the overall pooled estimate, after a lifestyle intervention. WMD—weighted mean difference; CI—confidence intervals; SD—standard deviation.

**Figure 3:**
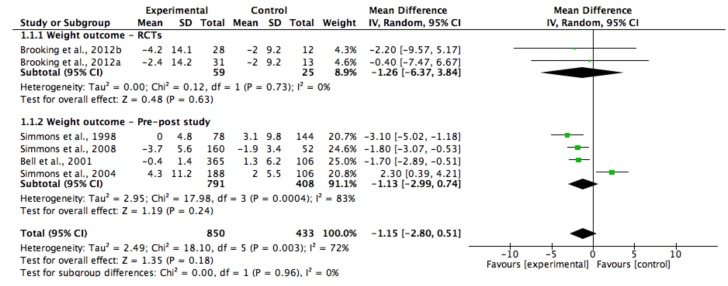
Forest plot showing the mean weight change and the overall pooled estimate, after a lifestyle intervention. One study had two different diet intervention groups (32). Brooking et al., 2012a [35] had an intervention group that emphasised a fiber rich carbohydrate and fat reduction (HCHF) diet; Brooking et al., 2012b [35] had the intervention group that utilised a high protein (HP) diet.

**Figure 4:**
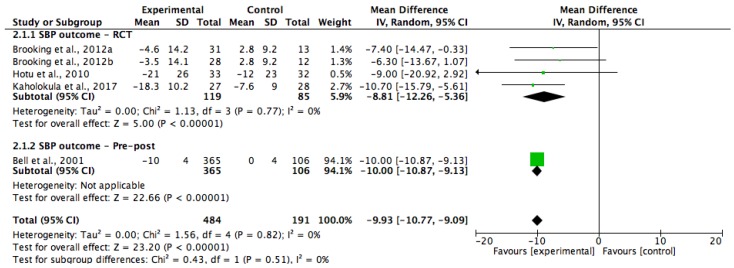
Forest plot showing mean systolic blood pressure (SBP) change and the overall pooled estimate after a lifestyle intervention. One study had two interventions groups (32). Brooking et al., 2012a [35] = intervention group that emphasised on a fiber rich carbohydrate and fat reduction (HCHF) diet; Brooking et al., 2012b [35] = the intervention group that utilised a high protein (HP) diet.

Two of the studies were included in a meta-analysis for HbA1c (Figure 2) [31,34], five of the studies were for weight (Figure 3) [29,30,32,33,35] and four of the studies were for systolic blood pressure (SBP) (Figure 4) [29,30,34,35]. Five of the study groups however, were analysed in the meta-analysis for SBP, and six of the groups were analysed in the meta-analysis for weight, as one of the included study’s had two intervention groups [35]. The pooled data showed that the lifestyle interventions had resulted in non-statistically significant reductions in HbA1c (WMD, −0.38%; 95% Cl, −1.15 to 0.39; and *p* = 0.33) and weight (WMD, −1.15 kg; 95% Cl, −2.80 to 0.51; and *p* = 0.18). The I^2^ statistic indicated that heterogeneity was present among the studies that had reported on HbA1c and weight (I^2^ = 58% and 72%, respectively). There was a statistically significant reduction in SBP in the lifestyle intervention group compared with the control group (WMD, −9.93 mmHg; 95% Cl, −10.77 to −9.09; and *p* < 0.00001), and the analysis had revealed no statistical heterogeneity (I^2^ = 0%) among the four studies.

#### 3.4.2. Narrative FindingsBody Mass Index (BMI)

##### Body Mass Index (BMI)

All of the eight included studies measured the BMI at baseline. One reported a decrease in BMI of >1.18 kg/m^2^ in the intervention groups over 24 weeks [35], and three found no significant change in BMI post-intervention [30,31,33]. The remaining four studies did not report on the follow-up BMI data [12,29,32,34].

##### Waist Circumference

Five of the studies had reported on changes in waist circumference [12,30,31,33,35]. Of these five studies, three had reported a reduction in waist circumference, between 0.7 cm [30] and 4.0 cm [33], in the intervention group [30,33,35]. No change was noted in one [31] and a non-significant waist circumference increase of +6 ± 8 cm was reported in another study [12].

## 4. Discussion

This review, for the first time, systematically identified and reviewed the available evidence on the impact of lifestyle interventions in the prevention and management of type 2 diabetes, specifically in Polynesian People, namely, a population with a high prevalence of obesity and type 2 diabetes. There were limited studies (*n* = 8) that were identified. The included studies provided evidence that lifestyle interventions could be effective in improving SBP and weight, but not glycaemic outcomes. However, we would advise caution when interpreting this conclusion because of limitations in the study design and reporting of studies, and small samples sizes (ranging between 55 [29] and 471 [30]).

The selected outcome measures and reporting varied across the studies and restricted the amount of data that could be pooled in the meta-analyses. A review and meta-analysis by Umpierre et al. [36] found that the combination of dietary and physical activity interventions had resulted in greater reductions in HbA1c and weight loss, compared with diet or physical activity alone, in people with diabetes. Other highly successful clinical trials, such as the USA Diabetes Prevention Program (DPP) and the Finnish Diabetes Prevention Study (DPS), included 3234 (20 of these were Pacific People) and 522 people with impaired glucose tolerance, respectively. These studies reported a ~6.7 kg and ~4.2 kg weight loss after an average of 2.8 years and 1 year, respectively, after an intensive lifestyle intervention (both through physical activity and diet) [23,24]. In the current review, the overall weight loss of the included studies ranged from ~0 to ~4.2 kg. The potential reasons for the lesser amount of weight loss that was reported in some of the community-based interventions that were included in this review, might have been as a result of the inclusion of motivated as well as less motivated community members (who joined to be part of a family/community activity), compared with the clinical trials, such as the DPP and DPS, where the participants were highly motivated study volunteers. In addition, the shorter duration of the interventions (ranging from 24 weeks to 2 years) and the smaller sample sizes might have resulted in less weight loss. Furthermore, all of the studies that were in this review reported the weight loss as mean changes, not the total number of participants that actually lost weight (which required larger numbers in order to achieve adequate power) [37]. Notably, the U.S. DPP suggested that there was a 16% reduction in imminent diabetes incidence, for every kilogram of weight that was lost, which was also adequate in order to prevent other non-communicable diseases, such as cardiovascular disease [19,22,38]. The overall weight loss of the included studies was 1.18 kg, therefore this review showed promise in terms of weight reduction and a consequent reduction in diabetes risk. Overall, the impacts of these trials on outcomes could be sustainable over a period of many years. For example, the Da Qing study showed that lifestyle interventions could have a long term impact on diabetes risk factors, with the participants in the intervention groups having shown a reduced annual diabetes incidence of 7%, compared with the11% in the control group, over a 20-year follow-up [39].

The systolic blood pressure was measured in most of the included studies. The pooled data resulted in a reduction of SBP by 9.93 mmHg. These findings were consistent with a previous meta-analysis by Dickinson et al., which was carried out in adults with a SBP of >140 mmHg that participated in a lifestyle intervention, which found a 5.0 mmHg reduction in SBP [40]. Similarly, these findings were also consistent with a larger lifestyle intervention trial on the reduction of cardiovascular risk factors, which found that lifestyle interventions had resulted in a systolic blood pressure reduction of 5.33 mmHg [41]. In this review, most of the individual studies had significant SBP reductions of between 4–21 mmHg. The highest reduction (21 mmHg) was noted in one study, where participants also had their anti-hypertensive medications adjusted by clinicians during the intervention [34], which was likely to have contributed to a greater reduction in SBP. Overall, these were promising findings, since a reduction of 5 mmHg in SBP could contribute to a 15% reduction in cardiovascular outcomes and reduce the incidence of stroke by 27% [29,42].

Many communities were enthusiastic about changing their diabetes status, however, most were reluctant to enrol in the experimental studies where they might not have received the intervention (if allocated to a control group), or they dropped out because of this same reason [33,43]. For example, in one study [33], 67% of participants that were referred for positive diabetes screening took an oral glucose tolerance test (OGTT) in the intervention group, compared with only 15% in the control group. As a result of the ethical issues when conducting lifestyle interventions in communities that are at high risk of chronic diseases, such as type 2 diabetes, researchers should be recommended to use culturally tailored interventions and more inclusive, but rigorous, study designs, such as the stepped-wedge trial and cross over designs, where all of the participants would receive the intervention at different stages during the study.

### Limitations and Recommendations for Future Practice

This review was limited, as the included studies had varying designs and health outcomes, as well as varying and short follow-up periods of between 24 weeks and 2 years. Therefore, heterogeneity might have been expected. For example, in two studies [12,33], the exact same intervention was delivered but very different results on health outcomes were achieved, possibly because of the differences in the population that were recruited. One of these studies included Samoan participants [33] and resulted in improved health outcomes compared with the other study, which included Tongan participants [12] in whom the participation rates were lower and revealed that there was no health impact after the intervention. Where data was provided, the recruitment processes were lengthy (between 15 to 22 months), which highlighted some of the complexity and difficulties in conducting community-based intervention programs [44]. Future studies would need to consider the time and effort that would be required in order to effectively recruit from such target populations. Additionally, the intensity, frequency, and duration of the physical activity that was undertaken in the interventions were poorly reported overall, and the exercise session attendance was recorded only in a few studies. The information on dietary content that was provided was also poorly reported, and none of the studies had utilized the intervention fidelity checks. The implementation of fidelity measurements in such community-based interventions was essential to determine whether the program adhered to the intended plan, and assisted in assessing the intervention efficacy [45]. Therefore, more robust studies of longer duration, with larger sample sizes and intention to treat analysis would be required for more conclusive evidence on the effectiveness of lifestyle interventions in Polynesian communities.

Secondly, there were differences in the intervention effectiveness in the studies, potentially because the interventions were being delivered in different settings and by a wide range of providers. The included interventions were delivered through face-to-face contact, by either nurses/community health workers, researchers, or skilled professionals. A review by Ali et al. [46] reported on similar studies, and a trial by Tang et al. [47], found that trained community workers were as effective as skilled professionals in producing behaviour changes in ethnic minority groups. The use of community coaches or peer support provided invaluable social and emotional support to people with, and at risk of diabetes, as the participants could relate to the common experiences of their peers on the diabetes prevention and management support [47,48,49]. A trial by Heisler et al. also found that the peer support interventions were more cost-effective, as they required less resources and the participants had greater improvements in glycaemic outcomes, compared with the health workers and skilled professional diabetes programs [49]. The inclusion of volunteer community members in order to deliver interventions, therefore, seemed plausible and important for sustainability.

Thirdly, all of the studies were conducted among populations that were living in urban areas. Therefore, the findings might not have been generalizable to the Polynesian People living in non-urban/remote areas.

Finally, the study quality was generally low, rating as high/serious risk of bias in items across studies, largely because the assessors/participants were not blinded to the participants’ intervention groups and from a lack of reporting for confounders. These issues underlined some of the challenges that were faced when conducting community-based interventions [44]. None of the included RCTs mentioned allocation concealment or assessor blinding. Similarly, the included pre–post studies had unblinded assessors and did not adjust for potential confounders. These areas of bias would need to be addressed in future research, where possible. Realistically, the allocation concealment and participant blinding was impractical in the lifestyle interventions research. The existing Risk of Bias tools were rigid in these aspects, therefore, more flexible, yet still robust, Risk of Bias tools for community-based lifestyle interventions were much needed, given that the interventions of this nature showed much promise in terms of reaching a wider target group and for sustainability in diabetes research. A Risk of Bias tool that could accommodate the different aspects of the community- and population-based lifestyle interventions, which considered that these interventions took time and required considerable community engagement, was necessary. The changes in lifestyle behaviours must have happened before the resultant clinical changes occurred, which could take a considerably longer time. Future research should consider measuring not only physiological health outcomes, but it should also capture the processes of behaviour change, such as readiness to change and quality of life. This would be particularly important where long-term follow-ups were not possible.

## 5. Conclusions

This review and meta-analysis shows that lifestyle interventions that were adapted to the meet the needs of the community, resulted in modest improvements in health outcomes in Polynesian People with, and/or at risk of developing type 2 diabetes. However, this may not have been because of a lack of impact of interventions, but rather as a result of limitations in the study design, reporting, drop out, and sample size. The difficulty of undertaking this type of community-based research has been acknowledged. Producing even small effects on health outcomes can have significant impacts at community levels for people with, and at risk of type 2 diabetes. This review has also highlighted that there is currently a lack of adequate evidence on the lifestyle intervention in Polynesian People regarding diabetes prevention and management. Culturally acceptable community-based trials of lifestyle interventions with a longer follow-up, outside of New Zealand and the United States of America, are urgently needed in order to address the growing diabetes epidemic in this high risk population.

## Figures and Tables

**Table 1:** Characteristics of studies that are included in the systematic review. M—mean; SD—standard deviation; RCT—randomised controlled trials.

*Studies on Diabetes Prevention*							
Author, (Year), Country	Aim	Study Design	Duration	Study Population	Study Population Age (M ± SD); Gender (%), n = Study Participants	Intervention and Control Groups	Outcome Measures
Brooking et al. (2012) New Zealand [35]	To determine the effects of a fibre rich carbohydrate, fat reduction, or a high protein diet on body fat in Maori that are at risk of diabetes.	RCT	24 weeks	New Zealand Maori	Intervention (HCHF): 45.8 ± 12.75; Male (26%); *n* = 31. Intervention (HP): 38.9 ± 10.50; Male (29%); *n* = 28 Control: 35.9 ± 10.9; Male 36%; *n* = 25.	Intervention: Participants were randomised into two interventions groups (fibre rich carbohydrate and fat reduction [HCHF] group, and a high protein diet [HP] group). The participants received prescriptive dietary advice, such as meal options, and portion sizes specific to the allocated diet group, with cooking demonstrations and food shopping tours provided. The diet intervention was based on the recommendations of the European Association for the Study of Diabetes (EASD) and American Diabetes Association (ADA). Control group: continued with their usual diet and received dietary advice at end of the study.	Weight (kgs); waist circumference (cms); total fat mass (kg).
Kaholokula et al. (2017) USA (Hawaii) [29]	To assess the feasibility and efficacy of Hula, a traditional Hawaiian dance, in reducing blood pressure in the Native Hawaiians and Pacific Islanders (NHPI) community.	RCT	12 weeks	Native Hawaiians and Pacific Islander	Intervention:55 ± 10; Female (93%); *n* = 27 Control: 55 ± 12; Female (79%); *n* = 28	Intervention: participants received 3 h of hypertension education and two 60-min classes of hula instruction and training per week, over 12 weeks. Waitlist (control) group: participants did not receive any intervention materials and no contact was made during the time they were on the waitlist.	Blood pressure (mmHg); physical functioning (assessed by the 6-min walk test); health related quality of life (HRQL).
Bell et al. (2001) New Zealand (Auckland) [30]	To evaluate the impact of a 1-year nutrition and exercise intervention program in promoting weight loss in three Samoan church communities.	Quasi-experimental (pre-post)	12 months	Samoans	Intervention: 43.9 ± 13.7; Female (60%); *n* = 365 Control: 39.1 ± 13.2; Female (60%); *n* = 106.	Intervention: participants received informal nutrition education sessions 1 h long (a total of 31 sessions held) and aerobic sessions were incorporated into regular church activities (total of 170 aerobic sessions were conducted) delivered initially by trained instructors from Pacific Islands Heartbeat (PIHB) program. Control: no details provided on the control group	Weight (kg); BMI (kg/m^2^); blood pressure (mmHg); diet and physical activity (questionnaire).
Simmons et al. (1998) New Zealand (South Auckland) [33]	To investigate the impact of a 2-year diabetes awareness/exercise lifestyle programme among a Samoan church congregation at risk of diabetes.	Pre-post	2 years	Samoans	Intervention 37 ± 16; Female (66%); *n* = 67 Control: 35 ± 17; Female (61%); *n* = 115.	Intervention: The Samoan community’s diabetes educator presented four diabetes awareness sessions (video and flipcharts), which were later followed by exercise groups consisting of aerobics, walking, sports, and sitting exercises, which were delivered by a Samoan health worker that was trained as an aerobics instructor, which were held weekly for the first year and twice per week in the second year. The participants attended practical sessions on cooking demonstrations. Control: the usual care, received the intervention upon study completion.	Weight (kg).Waist circumference (cms); diabetes knowledge (validated questionnaire)
Simmons et al. (2004) New Zealand (South Auckland) [12]	To assess the impact of a 2-year diabetes risk reduction programme on weight and exercise in a Samoan and Tongan church congregation.	Pre-post	2 years	Tongans	Intervention group: 33 ± 13; Female (52%); *n* = 167. Control: 34 ± 13; Female (49%); *n* = 86.	Intervention: The intervention church used leaflets and videos that were translated to Tongan. The messages that were delivered covered topics on diabetes and its symptoms and complications; nutrition, which included cooking demonstrations; and exercises sessions that were delivered by a trained aerobics instructor. Control: the usual care, received intervention on study completion.	weight (kg); waist circumference (cms).
Simmons et al. (2008) New Zealand [32]	To evaluate whether the intensive lifestyle interventions were effective in preventing or delaying type 2 diabetes among Maori community.	Pre-post	6 months	New Zealand Maori	Intervention:47 ± 13; Male (34.4%); *n* = 160. Control: 50 (13); Male (40.4%); *n* = 52.	Intervention: participants received the intervention based upon 12 key diet and physical activity messages (adapted from the Maori diet and physical activity behaviours) and were delivered by a trained Maori Community Health Worker (MCHW). Control: the usual care, did not receive intervention on study completion.	Weight (kg).
*Studies on Diabetes Management*							
**Author, (Year), Country**	**Aim**	**Study Design**	**Duration**	**Study Population**	**Age (M ± SD)**	**Intervention and Control Groups**	**Outcome Measures**
DePue et al. (2013) USA [31]	To evaluate the effectiveness of a primary care nurse and community health worker team in improving diabetes management among American Samoa.	RCT	12 months	American Samoa	Intervention: 55 ± 12.5; Female (57%); *n* = 104. Control: 54 ± 12.9; Female (65%); *n* = 164.	Intervention: Participants were seen weekly, in a group meeting held by the nurse care manager and a CHW, if considered to be high risk participants, moderate risk and low risk participants were seen by a CHW monthly and every 3 months, respectively (the length of the meetings was not specified). The meetings were based on diabetes management covering eight topics of healthy eating, physical activity, medication use, healthy coping, monitoring and understanding of blood glucose, and blood pressure measurements. Blood sugar was measured on each encounter. Received a copy of “Four steps to control diabetes for life”. Usual care (control) group: To receive intervention after 12 months. Received one phone call at 6 months to promote study retention rates. Received a copy of “Four steps to control diabetes for life”, no further contact was made until the end of the intervention.	HbA1c (%); BMI (kg/m^2^); waist circumference (cms); blood pressure (mmHg).
Hotu et al. (2010) New Zealand (Auckland) [34]	To determine whether the community health-care assistants are more effective in achieving and maintaining BP targets in Maori and Pacific patients.	RCT	12 months	Maori	Intervention:63 ± 6.6; Female (45%); *n* = 33. Control: 60 ± 7.1; Female (47%) *n* = 32.	Intervention: Ongoing monthly education on the importance of medication compliance, dietary modification, exercise, and smoking cessation. Participants were visited monthly by nurse/ health care assistant. Anti-hypertensives were adjusted regularly by a physician using a stepwise protocol. Received a diabetes education package at the start of the intervention Control: no alterations were made in their lifestyle or medication.	SBP (mmHg);HbA1c (%); 24-h urine protein excretion (g/day); total cholesterol (mmol/L).

## Supplementary Materials

The following are available online at http://www.mdpi.com/1660-4601/15/5/882/s1, Table S1: EMBASE database search strategy, Table S2: Risk of bias summary for RCTs using Cochrane collaboration’s tool for assessing risk of bias, Table S3: Risk of bias summary for pre-post studies using Risk of Bias in Non-Randomised Studies of interventions (ROBINS-1) assessment tool, Table S4: Reasons for exclusion of studies at final screening.
