# Supplementary material for: Lifestyle Interventions for People with, and at Risk of Type 2 Diabetes in Polynesian Communities: A Systematic Review and Meta-Analysis
Source: Int J Environ Res Public Health. 2018 Apr 28;15(5):882. doi: 10.3390/ijerph15050882 (PMC5981921; doi:10.3390/ijerph15050882)
Supplement: Supplementary file 1 [file ijerph-15-00882-s001.pdf]

Supplementary materials

Table S1: EMBASE database search strategy

S1.	Diabetes or 'diabetes mellitus' or 'type 2 diabetes' or NIDDM
S2.	Pacific* or Pasifika* or Samoa* or Tonga* or Maori* or Tahiti* or 'cook island' or Hawaii* or Tuvalu* or Niue* or Tokelaua* or Polynesian
S3.	(S1 AND S2)
S4.	diet or exercise or 'physical activity' or Intervention or 'diabetes prevention' or 'diabetes management'
S5.	(S3 AND S4)
S6.	'randomised control trial* or RCT or pre-post or program or intervention
S7.	(S5 AND S6)

Table S2: Risk of bias summary for RCTs using Cochrane collaboration's tool for assessing risk of bias

	Random sequence generation	Allocation concealment	Blinding of personnel	Blinding of outcome assessment	Incomplete outcome data	Selective reporting	Overall risk of bias
Brooking et al., 2012	2	2	2	2	1	1	High
DePue et al., 2013	1	2	2	2	1	1	high
Hotu et al., 2010	2	2	2	2	1	1	High
Kaholokula et al., 2017	2	2	2	2	1	1	High

1 = yes meets criteria, low risk of bias

2 = no doesn’t meet criteria or unclear, high risk of bias

Table S3: Risk of bias summary for pre-post studies using Risk of Bias in Non-Randomised Studies of interventions (ROBINS-1) assessment tool

	**Bias due to confounding**	**Bias in selection of participants into the study**	**Bias in classification of interventions**	**Bias due to deviations from intended interventions**	**Bias due to missing data**	**Bias in measurement of outcomes**	**Bias in selection of the reported result**	**Overall ROB**
**Bell et al., 2001**	2	2	1	2	1	3	1	Serious
**Simmons et al., 1998**	3	1	1	2	3	3	1	Serious
**Simmons et al., 2004**	3	1	1	2	3	3	1	Serious
**Simmons et al., 2008**	3	2	1	2	3	3	1	Serious

1 = yes meets criteria, low risk of bias

2 = some information provided, moderate risk of bias

3 = no doesn’t meet criteria or no information provided, high risk of bias

Table S4: Reasons for exclusion of studies at final screening

Author (Year) Country	Title	Journal	Reason for exclusion
Beckham et al., (2008)<br><br>USA (Hawaii)	Diabetes Management : Utilising community Health workers in a Hawaiian/Samoan Population	Journal of health care for the poor and underserved; 19: 416-427	Mixed ethnicities (Hawaiians n=52.6%, Samoan n=12.1%, Filipino n=9.5%, Caucasian n=14.6%, Tongan n=2.6% and other 8.6%)
Biddle et al., (2011)<br><br>New Zealand	Randomised controlled trial of informal team sports for cardiorespiratory fitness and health benefit in Pacific adults	Journal of primary health care; 3(4): 269-277	The study was over four weeks.<br>The study does not meet the inclusion criteria of ≥3 month
Choong (2016)<br><br>USA	Clinical outcomes of a diabetes education program for patients with diabetes mellitus in the Micronesian community in Hawaii	Journal of research in pharmacy practice; 5(3): 205–211.	There was no comparison group.
DeJoy et al., (2013)<br><br>USA	Worksite translation of the diabetes prevention program: Formative research and pilot study results from FUEL your life	Health Promotion Practice; 14(4): 506-513	Mixed ethnicities (White 79% and others 21%)
Gellert, Aubert & Mikami (2010)	Ke 'Ano Ola: Moloka'I's community-based healthy lifestyle modification program	American journal of public health	Mixed ethnicities
Gillelson et al., (2007)<br><br>Marshall Islands	A pilot food store intervention in the Republic of the Marshall Islands	Pacific health dialog; 14(2): 43-53	Intervention <3 months and participants were of different ethnic group
Hirsch et al., (2002)<br><br>USA	A multifaceted intervention in support of diabetes treatment guidelines: a cont trial	Diabetes research and clinical practice; 58(1): 27-36	Mixed ethnicities (White >50%)
Ing et al., 2016<br><br>USA	Social support groups in the maintenance of glycemic control after community-based intervention	Journal of Diabetes research;	Mixed ethnicities (Hawaiian n=27, Micronesian n=16, Filipino n=2 and others n=2)
Kaholokula et al., (2012)	A family and community focused lifestyle program prevents weight regain in	Health Education & Behavior: The Official Publication Of The	Mixed ethnicities (Native Hawaiians n=75, Samoans

USA	Pacific Islanders: a pilot randomized controlled trial	Society For Public Health Education; 39(4): 386-395	n=16, Chuukese n=38, Filipinos n=9)
Kaholokula et al., (2013)<br><br>USA	Sociodemographic, behavioural and biological variables related to weight loss in Native Hawaiians and other Pacific Islanders	Obesity; 21(3): E196-203	No control group
Krebs et al., (2013)<br><br>New Zealand	A structured, group-based diabetes self-management education (DSME) programme for people, families and whanau with type 2 diabetes (T2DM) in New Zealand: An observational study	Primary care diabetes; 7(2): 151-158	No comparison/control group
Ma et al., (2013)<br><br>USA	Translating the diabetes prevention program lifestyle intervention for weight loss into primary care: A randomized trial	JAMA internal Medicine; 173(2): 113-121	Mixed ethnic groups (Non-Hispanic white n=78%; Latino/Hispanic n= 4.1%; Asian/Pacific Islander n=17.0%)
Mau et al., (2001)<br><br>USA	Mediators of lifestyle behaviour change in Native Hawaiians	Diabetes care; 24(10) 1770-1775	Mixed ethnic groups (Non-Hispanic white n=78%; Latino/Hispanic n= 4.1%; Asian/Pacific Islander n=17.0%)
Mau et al., (2010)<br><br>USA<br><br>Kaholokula et al., (2013)<br><br>USA	Translating the Diabetes Prevention Program in Native Hawaiian and Pacific Islander communities: the PILI 'Ohana Project<br><br>Translating the Diabetes Prevention Program in Native Hawaiian and Pacific Islander communities: the PILI 'Ohana Project	Progress in community health partnerships: research, education, and action; 4(1): 7-16<br><br>Translational behavioural medicine; 4(2): 149-159	No comparison/control group<br><br>No control group
McAuley et al., (2003)<br><br>New Zealand	Implementation of a successful lifestyle intervention programme for New Zealand Maori to reduce the risk of type	Asia Pacific journal of clinical nutrition; 12(4): 423-426	No comparison/control group

	2 diabetes and cardiovascular disease		
McElfish et al., (2015)<br><br>USA	Family model of diabetes education with a Pacific Islander community	The diabetes educator; 41(6): 706-715	No comparison/control group
Murphy et al., (2003)<br><br>New Zealand	A new approach to design and implement a lifestyle intervention programme to prevent type 2 diabetes in New Zealand Maori	Asia Pacific Journal of Clinical Nutrition; 12(4): 419-422	No comparison group
Nishita et al., (2013)	Empowered Diabetes Management: Life Coaching and Pharmacist Counseling for Employed Adults With Diabetes	Health Education & Behavior; 40(5):581-591	Mixed ethnicities (Native Hawaiian or Pacific Islander 34.74%, White 17.37%, Asian 35.79%)
Simmons & Voyle (2003)	Reaching hard-to-reach, high risk populations: piloting a health promotion and diabetes disease prevention programmes on an urban Marea in New Zealand	Health promotion international; 18(1): 41-50	No control group
Sinclair et al., 2013	Outcomes from a Diabetes Self-management Intervention for Native Hawaiians and Pacific People: Partners in Care	Ann.behav.med; 45: 24-32	Mixed ethnicities
Sukala et al., (2011)<br><br>New Zealand	South Pacific islanders resist type 2 diabetes: comparison of aerobic and resistance training	Journal of physical activity and Health; 10(5); 699-707	No control group
Townsend et al., (2016)<br><br>USA	The PILI@Work Program: a translation of the diabetes prevention program to Native Hawaiian-serving worksites in Hawai‘i	Translational behavioural medicine; 6(2): 190-201	No control group

Uehara et al., (2011)<br><br>USA	The Influence of Individualised Supports on the Self-Efficacy of Employed Diabetics in Hawai'i: Findings from a Mixed Method Study	Journal of Pacific Rim Psychology; 5(2): 65-74	Mixed ethnicities
Wang et al., (1999)<br><br>USA	Development of a Community-Based Diabetes Management Program for Pacific Islanders	The diabetes educator; 25(5): 738-746	No control group
Wilhide et al., (2008)<br><br>USA	Impact of behavioural adherence on clinical improvement and functional status in a diabetes disease management program	Disease management; 11(3): 169-175	Included participants with type 1 diabetes and <18 years old.
Wilson et al., (2010)<br><br>USA	FUEL your life: A translation of the diabetes prevention Program to worksites	American journal of health promotion: AJHP	Mixed ethnicities
Win et al., (2016)<br><br>Nauru and Solomon Island	Comparing metabolic control and complications in type 2 diabetes in two Pacific Islands at baseline and following diabetes care intervention	Journal of clinical an Translational endocrinology	No control group
